# Diagnostic Dilemma in a Case of Necrotizing Pneumonia With Negative Transbronchial Biopsy

**DOI:** 10.7759/cureus.38327

**Published:** 2023-04-30

**Authors:** Vashistha M Patel, Shreya V Patel, Jerry Grant, Allison Rogers

**Affiliations:** 1 Internal Medicine, Brookwood Baptist Health, Birmingham, USA

**Keywords:** bronchoscopic biopsy, amoxicillin-clavulanate, prevotella, necrotizing pneumonia, 16s rrna gene sequencing analysis

## Abstract

A 33-year-old male with a past medical history of asthma presented to the Emergency room with a three-day history of right-sided chest pain, productive cough with dark brown sputum, and shortness of breath. He was found to have right lower lobe consolidation consistent with acute pneumonia, and areas of non-homogenous density within the consolidation, suspicious of necrotizing pneumonia. Computed tomography (CT) of the chest with IV contrast revealed a large, irregular thick-walled cavitary mass involving the right middle lobe with surrounding ground glass cavitation. An extensive workup was negative, including a transbronchial biopsy. The case explains how a causative organism was detected.

## Introduction

Accurately identifying bacterial isolates is an essential task of clinical microbiology. Sometimes phenotypic methods, such as bacterial cultures, cannot identify the pathogen on certain instances. Gram-positive rods are routinely not identified with this technique. This is a time-consuming process, especially when attempting to identify a specific fungus or bacterium. A relatively new alternative to the traditional method is the sequencing of the 16S ribosomal RNA gene or rRNA gene. The 16S rRNA gene is a short section of prokaryotic DNA found in all bacteria and archaea. It contains conserved regions for primers to bind and amplify various fragments of the 16S rRNA gene [1,2]. These genetic fragments contain regions that have specific sequences unique to certain species of bacteria allowing for identification at a species level. This case presentation will highlight a scenario in which 16S rRNA Sequencing was used to successfully identify a pathogen causing necrotizing pneumonia after the traditional phenotypic methods failed.

## Case presentation

A 33-year-old male with a past medical history of asthma presented to the Emergency room with a three-day history of right-sided chest pain, productive cough with dark brown sputum, and shortness of breath. He denied any bloody sputum production and had no prior episodes of similar symptoms. He had a significant tobacco use history with 15 pack years of cigarette smoking. He denied any recent travel outside of the country. He worked as a custodian with environmental services at a local rehabilitation facility. On initial presentation, vital signs were within normal limits with a blood pressure of 129/79 mm Hg, oxygen saturation of 97% on room air, a pulse of 99 beats per minute, respiratory rate of 18 breaths per minute, and the patient was afebrile at 99.3F. A general exam revealed a thin male in mild distress. The lung exam revealed mild expiratory wheezes throughout bilateral lung fields with positive egophony heard in the right middle lung field. Initial lab work revealed a leukocytosis of 18,900/microliters with a neutrophilic predominance of 82.7% (Table 1).

**Table 1:** Complete Blood Count WBC: white blood cells; RBC: red blood cells; MCV: mean corpuscular volume; MCHC: mean corpuscular hemoglobin concentration; MCH: mean corpuscular hemoglobin; RDW: red cell distribution width

Laboratory Test	Result	Reference Range
WBC (L)	18.9	4.5 to 11.0 × 10^9^/L
Hematocrit (%)	41	41 - 53
MCHC (g/dl)	34.3	32.0 - 36.0
Hemoglobin (g/dl)	14.0	13.5 - 17.5
MCH (g/dL)	29.2	25.5 - 34.5
Platelets (x10^3^/uL)	240	130 - 450
RBC (L)	4.80	4.50 - 5.90
MCV (L)	85.0	81.0 - 99.0
RDW (%)	13.0	10.0 - 16.0
Immature Granulocytes (%)	0.6	<=1.0
Monocytes (%)	8.9	<=10.0
Basophils (%)	0.3	<=5.0
Lymphocytes (%)	7.2	20.0 - 51.0
Eosinophils (%)	0.3	0.0 - 4.0
Neutrophils (%)	82.7	42.0 - 75.0

The comprehensive metabolic panel was unremarkable (Table 2).

**Table 2:** Comprehensive Metabolic Panel BUN: blood urea nitrogen; ALT: alanine aminotransferase; AST: aspartate aminotransferase; ALP: alkaline phosphatase

Laboratory Test	Result	Reference Range
Sodium (mmol/L)	135	136 - 145
Potassium (mmol/L)	3.9	3.5 - 5.1
Chloride (mmol/L)	98	98 - 107
Bicarbonate (mmol/L)	25	21 - 31
BUN (mg/dL)	8	7 - 25
Serum Creatinine (mg/dL)	1.1	0.80 - 1.30
Glucose (mg/dL)	103	70 - 105
Anion Gap	12	5 - 15
Total Bilirubin (mg/dL)	1.1	0.3 - 1.0
Albumin (g/dl)	4.4	3.5 - 5.7
Total Protein (g/dl)	8.1	5.8 - 7.9
ALT (u/l)	20	7 - 52
AST (u/l)	22	13 - 39
ALP (u/l)	69	34 - 104
Calcium (mg/dL)	9.4	8.6 - 10.3
Serum Osmolality (MOSM/K)	269	275 - 295

**Figure 1:**
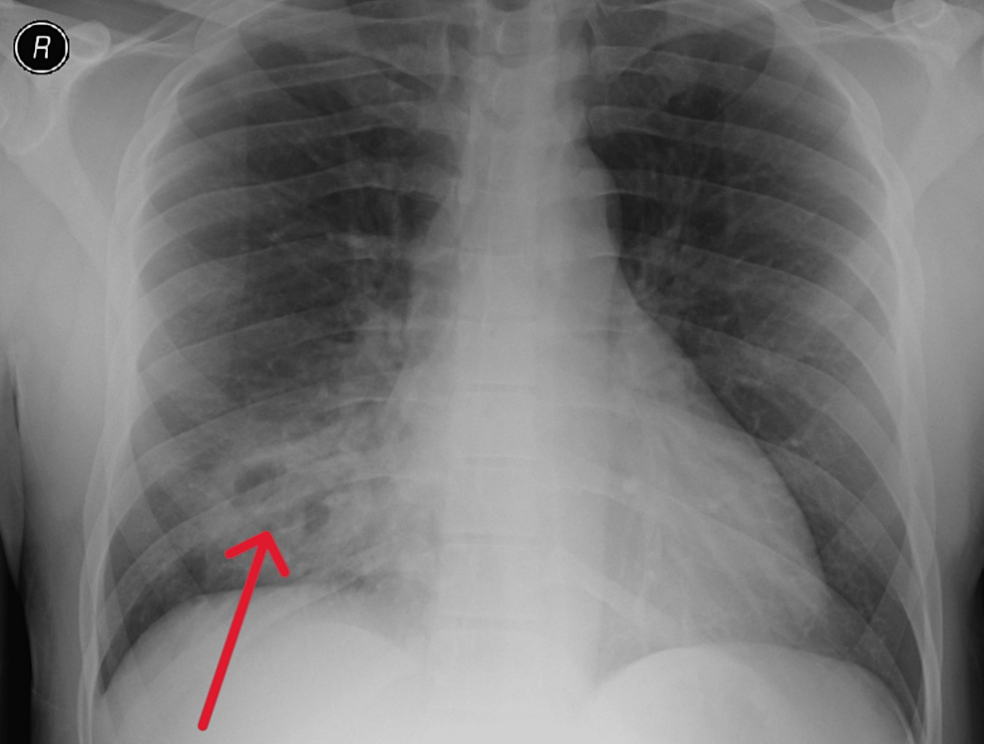
Chest X-ray The red arrow shows right lower lobe consolidation consistent with acute pneumonia with internal lucencies suggesting cavitary changes.

Plain radiographs of the chest showed a right lower lobe consolidation consistent with acute pneumonia with internal lucencies suspicious of necrotizing pneumonia (Figure 1).

**Figure 2:**
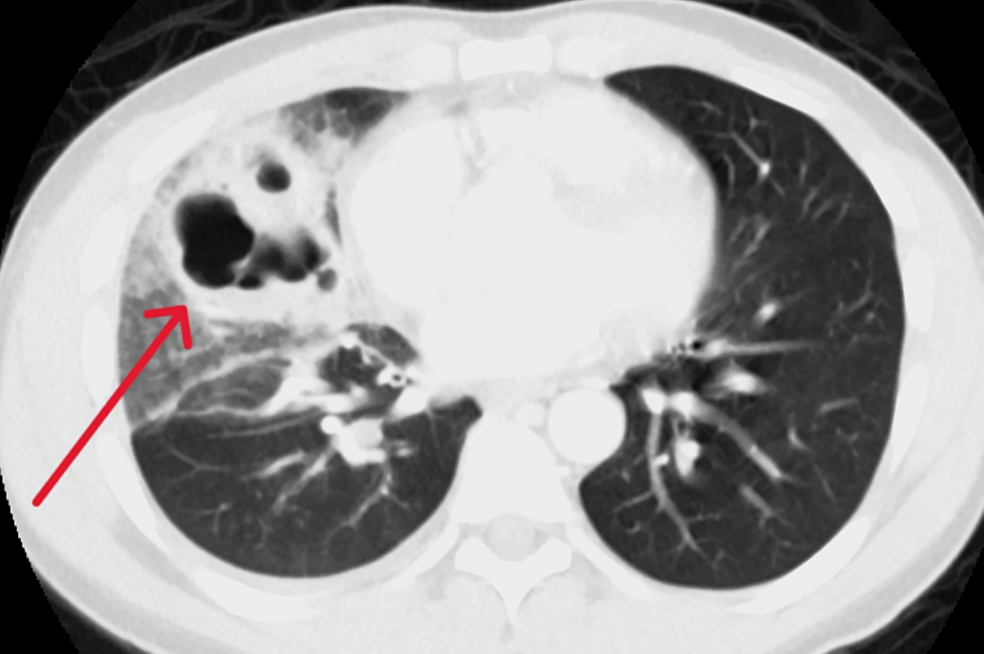
Computed tomography of chest The red arrow shows a large, irregular thick-walled cavitary mass involving the right middle lobe with surrounding ground glass consolidation with reactive mediastinal and hilar lymphadenopathy.

Computed tomography (CT) of the chest with IV contrast revealed a large, irregular thick-walled cavitary mass involving the right middle lobe with surrounding ground glass cavitation plus mediastinal and hilar lymphadenopathy (Figure 2).

**Figure 3:**
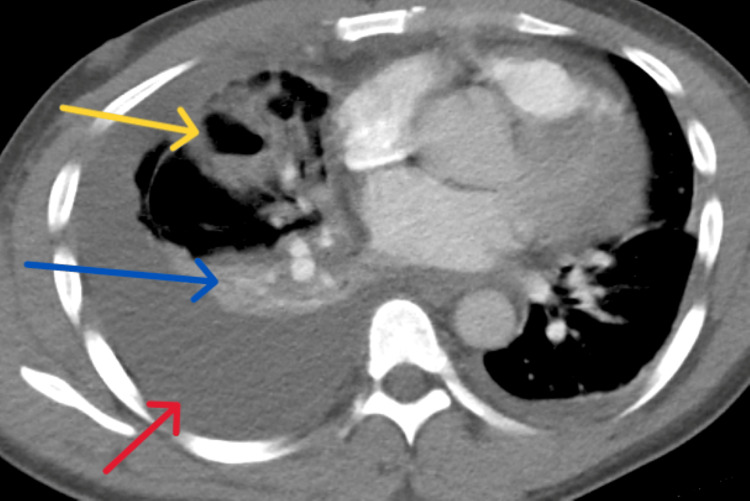
Computed tomography angiogram of chest The red arrow shows the development of the right-sided pleural effusion. The blue arrow shows near-complete compressive atelectasis of the right lower lobe. The yellow arrow shows the cavitary lesion.

The patient was initially started on broad-spectrum antibiotics with vancomycin and piperacillin-tazobactam. Due to no clinical improvement, subsequent computed tomography angiography (CTA) of the chest was obtained two days following admission revealing no interval change in the consolidation and newly found bilateral pleural effusions with compressive atelectasis (Figure 3).

Pulmonology was consulted for assistance with bronchoscopy and thoracentesis. Transbronchial biopsy specimens from the right middle lobe were negative for malignancy and revealed no organisms on the Gram, AFB, and fungal stains. Pleural fluid studies showed an exudative process, but the pathology was negative for malignancy. Culture data from blood, a biopsy from bronchoscopy, and pleural fluid remained negative. Workups for cryptococcus, blastomyces, and histoplasma were all negative. Vasculitis workup evaluating for granulomatosis with polyangiitis with C-ANCA panel was also negative. At this point in hospitalization, patients had been admitted for 12 days with a negative workup for the cause of the cavitary mass. On the 12th day of admission, a decision was made to transition to oral antibiotics with an extended course of amoxicillin-clavulanate. The patient was discharged with a close follow-up with the pulmonology and internal medicine clinic. Since the patient’s culture workup was negative, the decision was made to send bronchoscopy biopsy specimens for 16S rRNA sequencing to help delineate potential infectious etiology of the necrotizing consolidation and guide outpatient antibiotic therapy. The results of this test came back in approximately two weeks, revealing moderate growth of *prevotella oris*. The patient was started on Amoxicillin-clavulanate at the time of discharge and was recommended to remain on this to complete the extended course of antimicrobial therapy for a total of four weeks.

## Discussion

The purpose of this case report is to highlight the utility of 16S rRNA sequencing testing to identify infectious pathogens. In this case, this special testing successfully identified a single bacterial pathogen with *Prevotella oris.* *Prevotella oris* is a non-pigmented, Gram-negative, anaerobic bacterium that has been associated with serious infections. As the name implies, it is commonly found in the oral mucosa as well as the gut microbiota. It is known to cause a dentoalveolar abscess, spinal abscess, brain abscess, and anaerobic empyema. It has also been associated with bacteremia [1]. It has also been shown to be a pathogen associated with Lemierre’s syndrome, which is a rare form of sepsis secondary to oropharyngeal infection. The infection can then become disseminated, moving to the lungs and internal jugular vein, which can cause septic thrombophlebitis [2]. Discovery of the specific pathogen in our patient was important as it allowed for an appropriate choice of antibiotic therapy for the patient to remain on as an outpatient. Unfortunately, the patient had been lost to follow-up, and repeat imaging was unable to be obtained to monitor for the resolution of necrotizing pneumonia. 16S gene sequencing has been shown to better identify poorly described or rarely isolated organisms, and it can be used for the identification of mycobacterium that is very slow growing on typical culture media as well as other non-cultured bacteria [3, 4].

## Conclusions

Despite the accuracy of the testing, 16S rRNA gene sequencing lacks widespread use beyond the large research centers and reference laboratories due to technical and cost considerations. This test can be used as an additional tool to diagnose the organism when traditional methods fail and future consideration for this type of testing would be to transition this into a more convenient, easier-to-use test that can be performed at small clinical laboratories as well as develop ways to make this more affordable.
